# Staphylococcus aureus with reduced susceptibility to vancomycin isolated from a patient with fatal bacteremia.

**DOI:** 10.3201/eid0501.990118

**Published:** 1999

**Authors:** S S Rotun, V McMath, D J Schoonmaker, P S Maupin, F C Tenover, B C Hill, D M Ackman

**Affiliations:** United Hospital Medical Center (UHMC), Port Chester, New York, USA.

## Abstract

A Staphylococcus aureus isolate with reduced susceptibility to vancomycin was obtained from a dialysis patient with a fatal case of bacteremia. Comparison of the isolate with two methicillin-resistant S. aureus (MRSA) isolated obtained from the same patient 4 months earlier suggests that the S. aureus with reduced susceptibility to vancomycin emerged from the MRSA strain with which the patient was infected. Atypical phenotypic characteristics, including weak or negative latex-agglutination test results, weak or negative-slide coagulase test results, heterogeneous morphologic features, slow rate of growth, and vancomycin susceptibility (by disk diffusion test) were observed.


### *Staphylococcus aureus* with Reduced Susceptibility to Vancomycin Isolated from a Patient with Fatal Bacteremia

**Sharon S. Rotun,* Virginia McMath,* Dianna J. Schoonmaker, Peggy S. Maupin,† Fred C.Tenover,‡ Bertha C. Hill,‡ and David M. Ackman†**
*United Hospital Medical Center (UHMC), Port Chester, New York, USA;
†New York State Department of Health, Albany, New York, USA; and
‡Centers for Disease Control and Prevention (CDC), Atlanta, Georgia, USA

A *Staphylococcus aureus* isolate with reduced susceptibility to vancomycin was obtained from a dialysis patient with a fatal case of bacteremia. Comparison of the isolate with two methicillin-resistant *S. aureus* (MRSA) isolates obtained from the same patient 4 months earlier suggests that the *S. aureus* with reduced susceptibility to vancomycin emerged from the MRSA strain with which the patient was infected. Atypical phenotypic characteristics, including weak or negative latex-agglutination test results, weak or negative-slide coagulase test results, heterogeneous morphologic features, slow rate of growth, and vancomycin susceptibility (by disk diffusion test) were observed.

Three cases of infection by *Staphylococcus aureus* with reduced susceptibility to the glycopeptide antibiotics vancomycin and teicoplanin (glycopeptide intermediate *S. aureus*, GISA) have been reported from Japan, Michigan, and New Jersey (1,2). We report the third case in the United States of *S. aureus* with reduced susceptibility to vancomycin. We found that this GISA apparently emerged from a methicillin-resistant *S. aureus* (MRSA) isolate that had infected/colonized the patient during the 3 months before his death. This report highlights key phenotypic characteristics of interest to both clinicians and microbiologists.

The first (in New York State) *S. aureus* isolate with reduced susceptibility to vancomycin was obtained from the blood of a 79-year-old man with a history of coronary artery disease, obstructive pulmonary disease, hypothyroidism, and renal failure requiring chronic hemodialysis. Bacteremia developed in December 1997, and an MRSA was isolated from the patient's blood on December 31. He received vancomycin while in the hospital, improved clinically, and was discharged. He was readmitted to the hospital in January 1998 with an infected jugular catheter, which was removed. An MRSA isolate was obtained from the catheter on January 10, and MRSA was also isolated from the patient's blood. He improved clinically and was subsequently discharged on vancomycin therapy. From December 1997 to February 1998 he received 3.5 g of intravenous vancomycin, followed by a 7-day course of 3.5 g of oral ciprofloxacin. In March 1998, the patient was admitted to the dialysis center with signs of congestive heart failure and sepsis and was transferred to the hospital. He received vancomycin (1 g), ceftriaxone (2 g), and tobramycin (200 mg) but died the following day. Over the last 4 months of his life, the patient received 8.0 g of vancomycin for various infections or for surgical prophylaxis of thrombectomy repairs. An autopsy was not performed.

The vancomycin MIC of the isolate, obtained from blood collected before the administration of antibiotics on March 20 at the patient's last hospital admission, was determined by the hospital laboratory to be 8 µg/ml by Etest (3) and MicroScan conventional panels (Dade Behring, Inc., MicroScan). This MIC was confirmed by broth microdilution at the New York State Department of Health and the Centers for Disease Control and Prevention (4). The MRSA isolates obtained from the patient in December and January were susceptible to vancomycin by

Address for correspondence: Dianna J. Schoonmaker, Wadsworth Center, New York State Department of Health, 120 New Scotland Avenue, Albany, NY 12208, USA; fax: 518-486-7971; e-mail: djs03@health.state.ny.us.

broth microdilution (MIC <2 µg/ml) and Etest (3 µg/ml and 4 µg/ml, respectively). All three isolates appeared to be susceptible to vancomycin when tested by the disk diffusion method, with zones of 17 mm to 18 mm (5), and were susceptible to teicoplanin by broth microdilution (MIC 2-4 µg/ml). Broth microdilution and Etest results showed that in addition to vancomycin and methicillin, the GISA isolate is also resistant to penicillin, erythromycin, ciprofloxacin, and rifampin; the two MRSA isolates from December and January were also resistant to the latter four antibacterial agents, although at higher levels than the GISA isolate for penicillin (>32 µg/ml and 6 µg/ml, respectively) and erythromycin (256 µg/ml and 16 µg/ml, respectively). All three isolates were susceptible to clindamycin, trimethoprim/sulfamethoxazole, gentamicin, chloramphenicol, and tetracycline. Although the susceptibilities of the recently described GISA isolates to antimicrobial agents vary, all are resistant to clindamycin (6), whereas the GISA isolate was susceptible.

The GISA isolate has two colony types, one of which is smaller than the typical *S. aureus* colony. The MRSA obtained from the patient in January also demonstrated colony size heterogeneity, unlike the MRSA obtained in December, which had only the larger typical colony type. Coagulase activity of the GISA isolate was detected at 24 hours by tube test but was only very weakly detected by slide test (NYSDOH) or was negative (UHMC, CDC). Atypical (weakly positive [Staphyloslide, BBL] or negative [Staphaurex, Murex]) results were obtained by latex-agglutination tests.

As part of a contact investigation, cultures were obtained from the nares and hands of dialysis center and hospital employees, health-care aides, dialysis center patients, and family members. Nares of 45 persons and the hands of 19 of the 45 were cultured; 14 methicillin-susceptible *S. aureus* (MSSA) and two MRSA isolates (one from a nephrologist and one from a dialysis patient) were obtained. However, no other GISA isolates were found. There was no evidence that the GISA was spread to other patients or employees.

The *S. aureus* isolates obtained from the patient in December, January, and March, as well as the MRSA from the nephrologist and the dialysis patient, were compared by pulsed-field gel electrophoresis (PFGE). The three isolates from the patient had the same PFGE type (Figure). The isolates from the nephrologist and the dialysis patient (not shown) had PFGE types unrelated to the GISA type and unrelated to each other. This suggests that GISA emerged from the MRSA strain that had infected the patient in December, presumably as a result of vancomycin therapy. Although the patient was admitted to the hospital in March with a high fever and evidence of pneumonia and the GISA isolate was obtained from blood specimens during hospitalization, it was not possible to determine whether vancomycin resistance in this organism was responsible for the patient's death.

The third reported in the United States, this case of GISA is similar to previously reported cases. The patient described above had been infected with MRSA for several months and had received prolonged treatment with vancomycin. Laboratories should retain MRSA isolates because they may be valuable in outbreak investigations. As in this case, MRSA isolates, together with subsequent isolates, can be used in studying the emergence of glycopeptide resis-

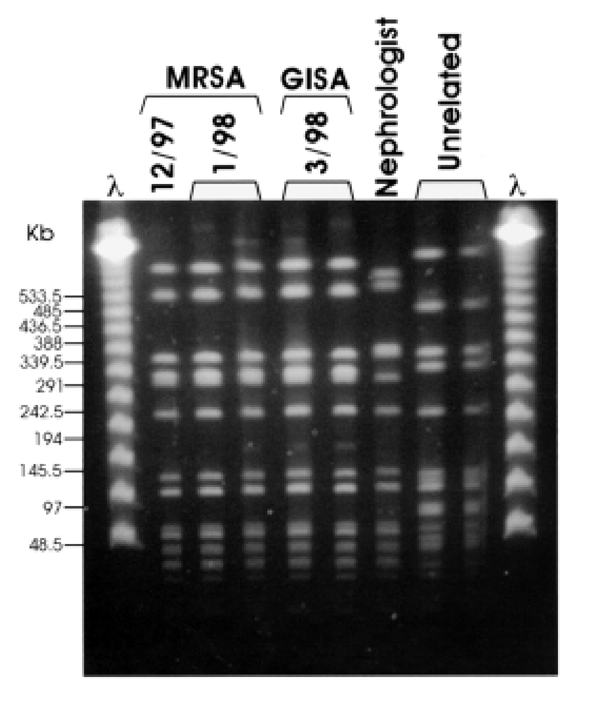

Figure. Pulsed-field gel electrophoresis (PFGE) profiles of *Sma*I digested DNA. Case patient MRSA isolates from December 1997 and January 1998, GISA isolate from March 1998. Nephrologist MRSA isolate from March 1998 and unrelated MRSA isolates.

tance in MRSA. The GISA isolate grew slowly, had heterogeneous morphologic features, weak or negative slide-coagulase test results, and weak or negative latex-agglutination test results. GISA appeared to be susceptible to vancomycin by disk diffusion, as was reported for the previous isolates (6). The disk diffusion test does not appear to be accurate for determining vancomycin susceptibility in these organisms. Microbroth dilution or the Etest method should be used instead. Atypical phenotypic characteristics have been reported for every other GISA, although not all isolates exhibited the same atypical characteristics (1,2,6,7). Laboratorians should be aware of these atypical characteristics so that GISA are not misidentified. Evidence for cell-wall reorganization has been reported for GISA isolates (1,7). These changes in cell-wall structure may be responsible for the atypical phenotypic characteristics and decreased susceptibility to vancomycin.

### Acknowledgments

We thank the staff of the United Hospital Microbiology Laboratory for their technical assistance and Larry Bopp, Dale Morse, Harry Taber, and Mehdi Shayegani for their assistance and support.

Dr. Rotun is microbiology manager at Our Lady of Mercy Health Care System, New York, NY. Her research interests include developing procedures to detect and monitor antimicrobial resistance in clinically important bacteria.

### References

1. Hiramatsu K, Hanaki H, Ino T, Yabuta K, Oguri T, Tenover FC. Methicillin-resistant *Staphylococcus aureus* clinical strain with reduced vancomycin susceptibility. J Antimicrob Chemother 1997;40:135-6.
2. *Staphylococcus aureus* with reduced susceptibility to vancomycin—United States. MMWR Morb Mortal Wkly Rep 1997;46:765-6.
3. Etest package insert. AB Biodisk North America., Piscataway, NJ. 1996.
4. National Committee for Clinical Laboratory Standards. Methods for dilution antimicrobial susceptibility tests for bacteria that grow aerobically. 4th ed. Vol 17, no. 2, Approved Standard M7-A4, National Committee for Clinical Laboratory Standards, Wayne, PA. 1998.
5. National Committee for Clinical Laboaratory Standards. Performance standards for antimicrobial disk susceptibility tests. 6th ed. Vol. 17, no. 1. Approved Standard M2-A6, National Committee for Clinical Laboratory Standards, Wayne, PA. 1998.
6. Tenover FC, Lancaster MV, Hill BC, Steward CD, Stocker SA, Hancock GA, et al. Characterization of Staphylococci with reduced susceptibilities to vancomycin and other glycopeptides. J Clin Microbiol 1998;36:1020-7.
7. Daum RS, Gupta S, Sabbagh R, Milewski WM. Characterization of *Staphylococcus aureus* isolates with decreased susceptibility to vancomycin and teicoplanin: isolation and purification of a constitutively produced protein associated with decreased susceptibility. J Infect Dis 1992;166:1066-72.

